# A Case of Paraquat Poisoning Presenting With Spontaneous Pneumothorax and Pneumomediastinum

**DOI:** 10.7759/cureus.11943

**Published:** 2020-12-07

**Authors:** Debananda Sahoo, Nilanjan Kar, Sujata Devi, Anupam Dey, Dhriti Sundar Das

**Affiliations:** 1 Internal Medicine, All India Institute of Medical Sciences, Bhubaneswar, IND; 2 General Medicine, All India Institute of Medical Sciences, Bhubhaneswar, IND; 3 General Medicine, All India Institute of Medical Sciences, Bhubaneswar, IND; 4 Medicine, All India Institute of Medical Sciences, Bhubaneswar, IND; 5 General Medicine, All India Institute of Medical Sciences, Bhuwaneswar, IND

**Keywords:** paraquat, pneumothorax, spontaneous pneumomediastinum, pulmonary fibrosis, reactive oxygen species

## Abstract

Paraquat (1,1’-dimethyl-4,4’-dipyridylium) is a liquid herbicide associated with accidental and suicidal ingestion, leading to fatal toxicity. It can lead to multiple organ dysfunction, including metabolic acidosis, acute kidney and liver injury, pulmonary fibrosis, and acute respiratory distress syndrome (ARDS). Very rarely, this can present with spontaneous pneumothorax or pneumomediastinum or both, which are poor prognostic markers with a mortality rate of almost 100%. Here, we present a young male presenting with paraquat poisoning followed by the development of both pneumothorax and pneumomediastinum and death from respiratory failure. Paraquat poisoning should always be considered in the differential diagnosis in patients presenting with spontaneous pneumothorax or pneumomediastinum in places with high paraquat poisoning prevalence.

## Introduction

Spontaneous pneumothorax and pneumomediastinum are usually defined as the presence of free air in the pleural cavity and the mediastinal structures, respectively, without any apparent precipitating cause or any underlying pulmonary disease. Around one in 12,000 cases of hospital admission presents with this [1], and it most frequently occurs in young, healthy adults without any underlying severe pulmonary disease [2]. Paraquat (1,1’-dimethyl-4,4’-dipyridylium) is a liquid herbicide associated with accidental and suicidal ingestion, leading to fatal toxicity [3]. It exerts its effect by generating reactive oxygen species (ROS), subsequently leading to the destruction of the cell membrane. Along with local corrosive damage to the oral cavity and gastrointestinal (GI) mucosa, it can cause multiple organ dysfunction, including metabolic acidosis, acute kidney and liver injury, pulmonary fibrosis, and acute respiratory distress syndrome (ARDS). Very rarely, this can present with spontaneous pneumothorax or pneumomediastinum or both. Paraquat poisoning has the highest mortality rate, accounting for 13% of all deaths occurring due to various poisoning [4].

## Case presentation

**Figure 1:**
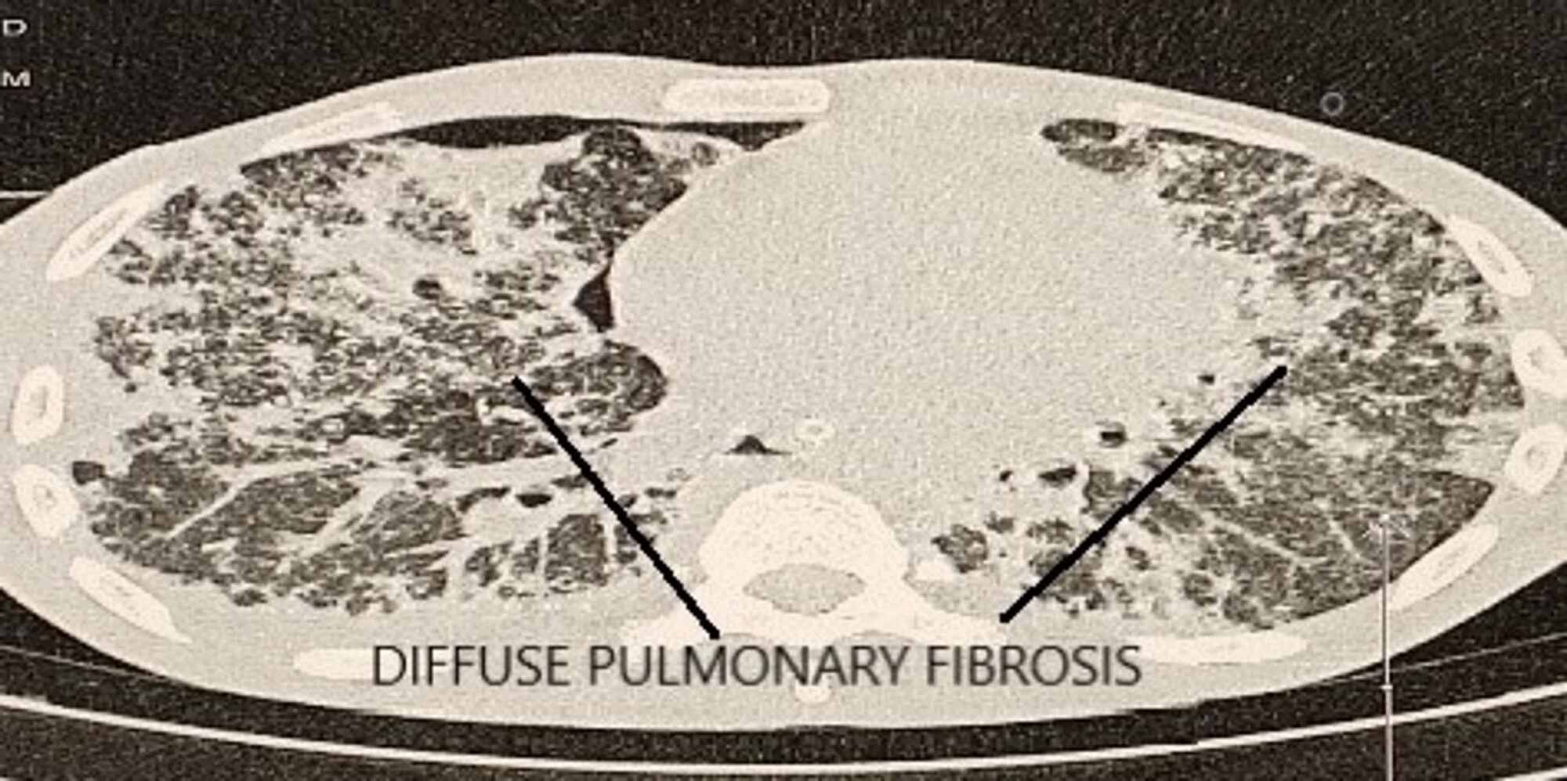
Diffuse fibrotic changes in bilateral lung fields

**Figure 2:**
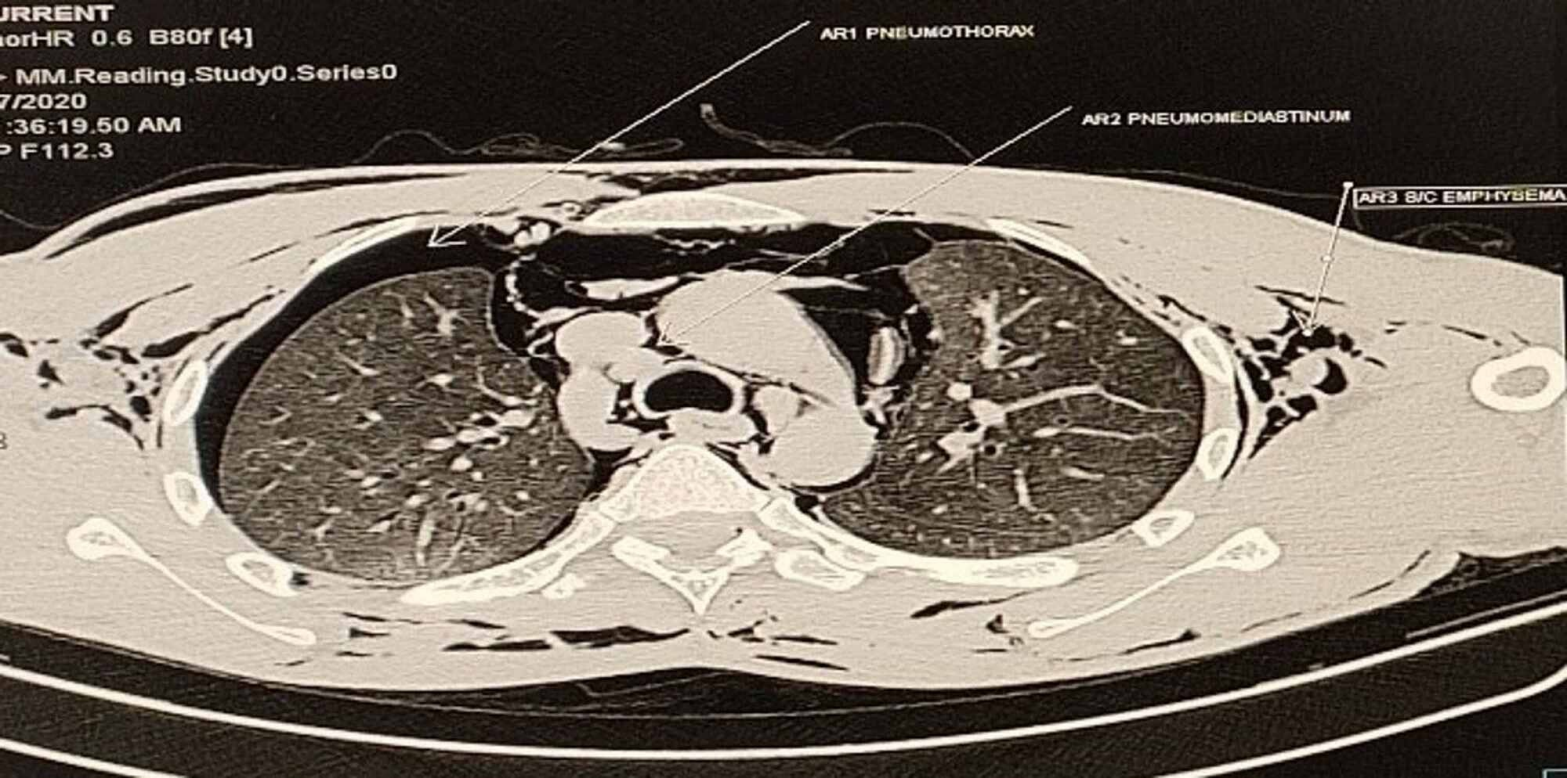
CT thorax showing pneumothorax, pneumomediastinum, and subcutaneous emphysema CT: computed tomography

**Figure 3:**
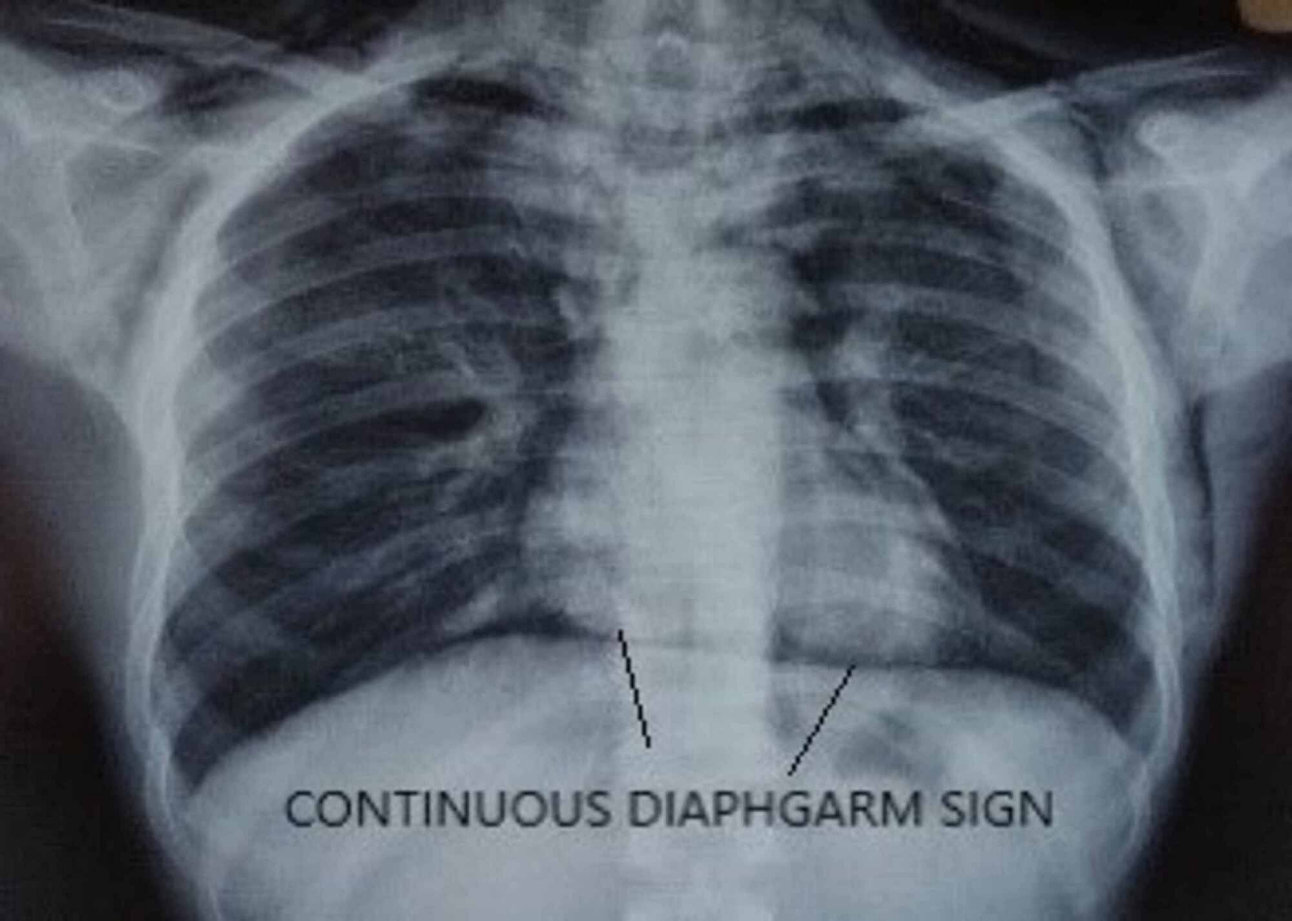
Continuous diaphragm sign in pneumomediastinum

A 22-year-old male patient presented to the emergency department (ED) of All India Institute of Medical Science, Bhubaneswar, with an alleged history of intentional consumption of approx. Forty (40) ml of 24% paraquat compound three days before the presentation. After consumption, he had several episodes of vomiting for which he underwent gastric lavage followed by conservative management with activated charcoal, intravenous fluids, antiemetics, and proton pump inhibitors (PPI) in a local hospital. He presented to the AIIMS, Bhubaneswar, ED three days after the incident with complaints of abdominal pain with bloody diarrhea, difficulty in deglutition, and hoarseness of voice for the last three days. On presentation in the ED, he was conscious and oriented, with stable vitals. Examination revealed oral mucosal congestion, ulceration with sloughing, and diffuse subcutaneous emphysema over the neck and chest. Upon initial evaluation, among his baseline laboratory parameters, total leukocyte count (TLC), liver function test (LFT), and electrolytes were within normal limits, with elevated urea/creatinine level: 86/2.16 mg/dl (0.5-1.2/15-40 mg/dl). Radiological investigations showed diffuse fibrotic changes in both lung fields (Figure 1), with bilateral pneumothorax (Right > Left) and pneumomediastinum as evidenced by computed tomography (CT) thorax (Figure 2) and X-ray chest (continuous diaphragm sign) (Figure 3).

There was no esophageal rupture by barium swallow study. The patient was managed conservatively with intercostal drainage tube placement on the right side with adequate hydration, PPI, liquid sucralfate, and empirical antibiotics. Despite the initial normalization of his renal parameters, he subsequently developed septicemia with the growth of Pseudomonas aeruginosa in blood culture and neutrophilic leukocytosis: 20×103/mm^3^ (4-11 × 103/mm^3^). He was started with antibiotics as per the culture-sensitivity report. But within the next two to three days, he again developed acute kidney injury, hepatopathy in the forms of raised bilirubin level (total bilirubin/direct bilirubin: 3.1/1.8 mg/dl) and liver enzymes (aspartate transaminase/alanine aminotransferase: 106/134 U/L) and increasing respiratory distress for which he was planned to be shifted to the intensive care unit (ICU) for better ventilatory management. Unfortunately, he succumbed to death on day eight of admission because of pulmonary fibrosis leading to respiratory failure.

## Discussion

Poisoning by the herbicide paraquat is a common cause of self-poisoning in vast parts of South-East Asia, including India. Because of the lack of effective treatment and inherent toxicity, the case fatality rate hovers around 50% to 90% [5]. Paraquat poisoning exerts its local and systemic manifestations by generating superoxide, hydrogen peroxide, and hydroxyl radicals in the form of oral, tongue, and GI mucosal ulcerations, liver and kidney dysfunction, and severe respiratory failure, as in the present described case. The lethal dose of paraquat for humans is around 30 mg/kg, which is equivalent to 8-10 mL of the 24% solution sold commercially [6].

In the lung, both type 1 and type 2 alveolar epithelial cells uptake the paraquat by an active energy-dependent polyamine uptake pathway leading to a very high concentration of paraquat in the lung parenchyma (10-20 times greater than in plasma) [7]. It causes ROS-mediated destruction of type 1 and type 2 pneumocytes. Damage to type 1 pneumocytes impairs oxygenation and capillary exchange; whereas destruction of type 2 pneumocytes leads to increased surface tension and subsequent fluid accumulation, causing pulmonary edema, and hemorrhage. Pathological healing sets in, with a response to the accumulation of inflammatory cells, cytokine release, and myofibroblast-mediated collagen deposition with the subsequent development of early lung fibrosis.

Paraquat-induced pneumothorax and pneumomediastinum were first described in 1990 by Daisley and Barton [8]. Their proposed mechanism was the initiation of this pathological repair at the alveolar level with interstitial widening, alveolar congestion, collagen deposition, and microthrombi formation, which leads to the early development of secondary pulmonary hypertension and an obvious consequence of pneumothorax and pneumomediastinum called “Daisley Barton syndrome” [9]. However, the most accepted mechanism was delineated by Macklin. He proposed that the destruction of type 2 pneumocytes causes secondary atelectasis, rupture, and emphysematous changes due to a rise in surface tension and the subsequent formation of peripheral or subpleural bullae that rupture, causing pneumothorax and escape of the gas to dissect along vascular sheaths and connective tissue planes in the mediastinum, causing pneumomediastinum [10].

Zhou et al. showed that early pneumomediastinum within eight days is a specific predictor of mortality in paraquat poisoning [11], indicating abysmal prognosis, with a mortality rate of almost 100% [12], as it happened in our patient. Early activated charcoal hemoperfusion with continuous venovenous hemofiltration in paraquat poisoning might prolong survival time by delaying systemic complications; however, a reduction in the mortality rate is uncertain and controversial. Patients developing acute renal failure should undergo hemodialysis. Paraquat-induced lung injury can be aggravated by oxygen therapy [13], hence this should be avoided as much as possible.

There is no specific effective antidote available for paraquat. A few case reports had shown N-acetylcysteine, corticosteroids, cytotoxic agents, antioxidants (high-dose vitamin C or E), nitric oxide supplement, or paraquat antibodies were used in the treatment of paraquat poisoning with varied outcomes. In moderate-to-severe cases of paraquat poisoning, corticosteroid and cyclophosphamide combination therapy may offer some benefits by preventing ongoing inflammation and pulmonary fibrosis [14]. The role of lung transplantation after paraquat poisoning is also supported by a few case reports [15].

## Conclusions

Pneumothorax and pneumomediastinum in a patient with paraquat poisoning is a less uncommon but underdiagnosed finding. It has a high index of early mortality, hence reasonable clinical suspicion is always warranted. In places where paraquat poisoning is quite prevalent, it should still be considered in the differential diagnosis of patients presenting with unexplained pneumothorax, pneumomediastinum, or ARDS.
